# Supplementary material for: Structural basis of human NOX5 activation
Source: Nat Commun. 2024 May 11;15:3994. doi: 10.1038/s41467-024-48467-y (PMC11088703; doi:10.1038/s41467-024-48467-y)
Supplement: Supplementary file 1 — Supplementary Information [file 41467_2024_48467_MOESM1_ESM.pdf]

Structural Basis of Human NOX5 Activation

Supplementary Information

Supplementary Figures 1-11

Supplementary Tables 1-3

Supplementary Figures

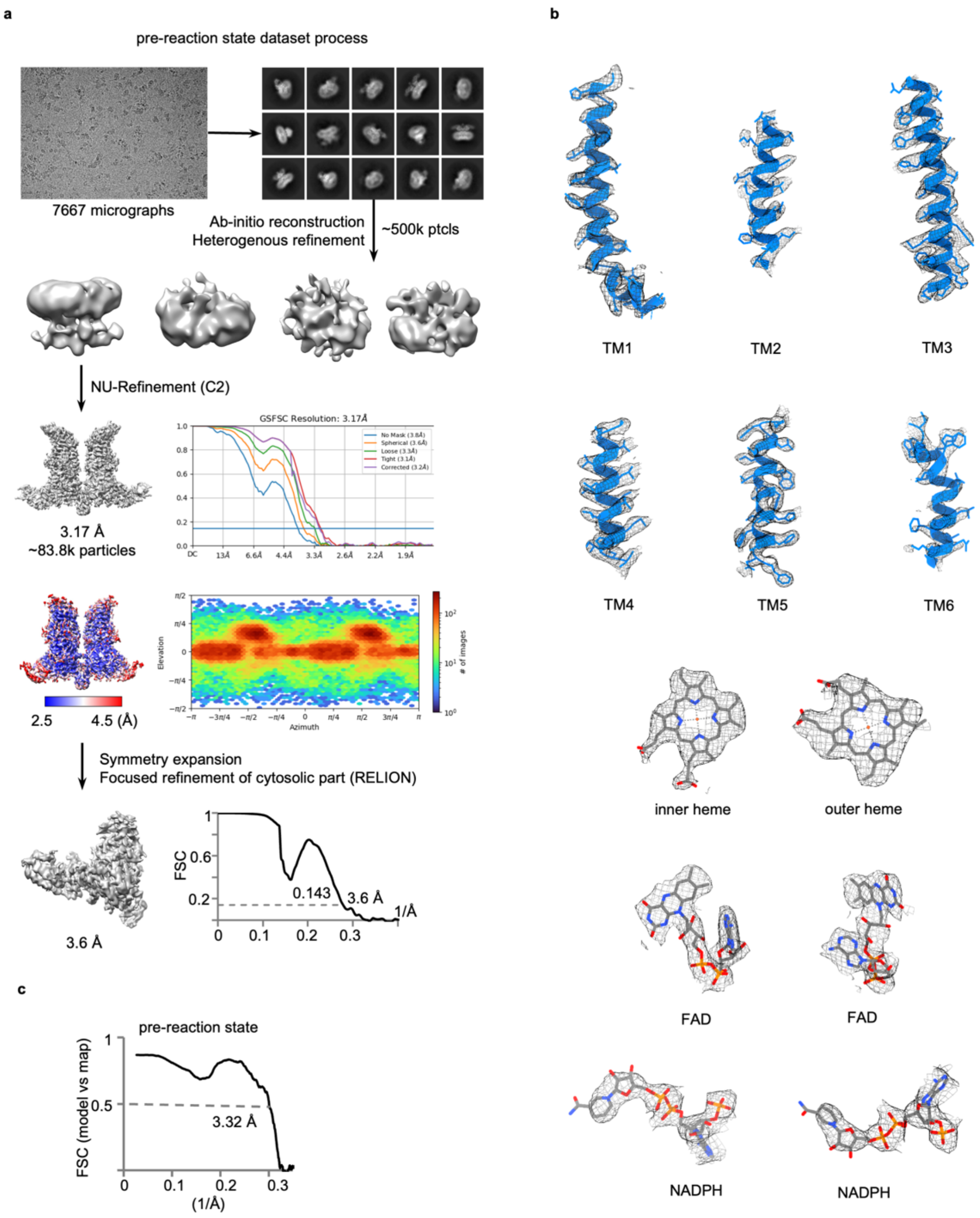

Supplementary Figure 1 Cryo-EM image analysis of NOX5 in pre-reaction state and representative cryo-EM density maps. **a.** Cryo-EM data processing flow of NOX5 in pre-reaction state (with NADPH and EGTA). **b.** The cryo-EM densities and models of transmembrane helices

and small molecules. **c.** Fourier shell correlation (FSC) curves between model and map of NOX5 in pre-reaction state.

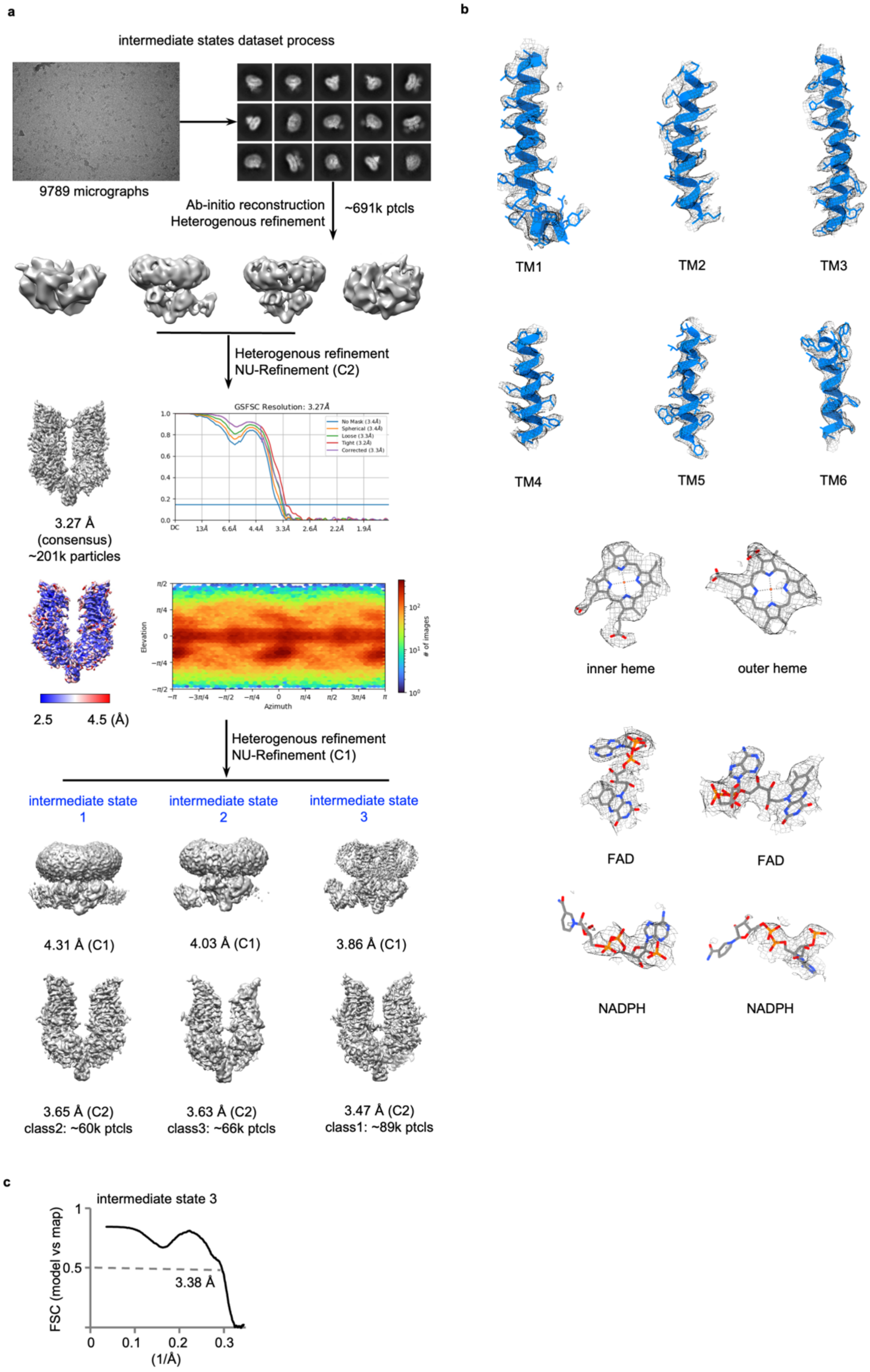
a
intermediate states dataset process
9789 micrographs
Ab-initio reconstruction
Heterogenous refinement
~691k ptcls
Heterogenous refinement
NU-Refinement (C2)
GSFSC Resolution: 3.27Å
3.27 Å
(consensus)
~201k particles
Elevation
Azimuth
# of images
2.5
4.5 (Å)
Heterogenous refinement
NU-Refinement (C1)
intermediate state 1
intermediate state 2
intermediate state 3
4.31 Å (C1)
4.03 Å (C1)
3.86 Å (C1)
3.65 Å (C2)
class2: ~60k ptcls
3.63 Å (C2)
class3: ~66k ptcls
3.47 Å (C2)
class1: ~89k ptcls
b
TM1
TM2
TM3
TM4
TM5
TM6
inner heme
outer heme
FAD
FAD
NADPH
NADPH
c
intermediate state 3
FSC (model vs map)
1
0.5
3.38 Å
0
0.1
0.2
0.3
(1/Å)

Supplementary Figure 2 Cryo-EM image analysis of NOX5 in intermediate states and representative cryo-EM density maps. **a.** Cryo-EM data processing flow of NOX5 in intermediate states (with NADPH and Ca^{2+}). **b.** The cryo-EM densities and models of transmembrane helices and small molecules (intermediate state 3). **c.** Fourier shell correlation (FSC) curves between model and map of NOX5 in intermediate state 3.

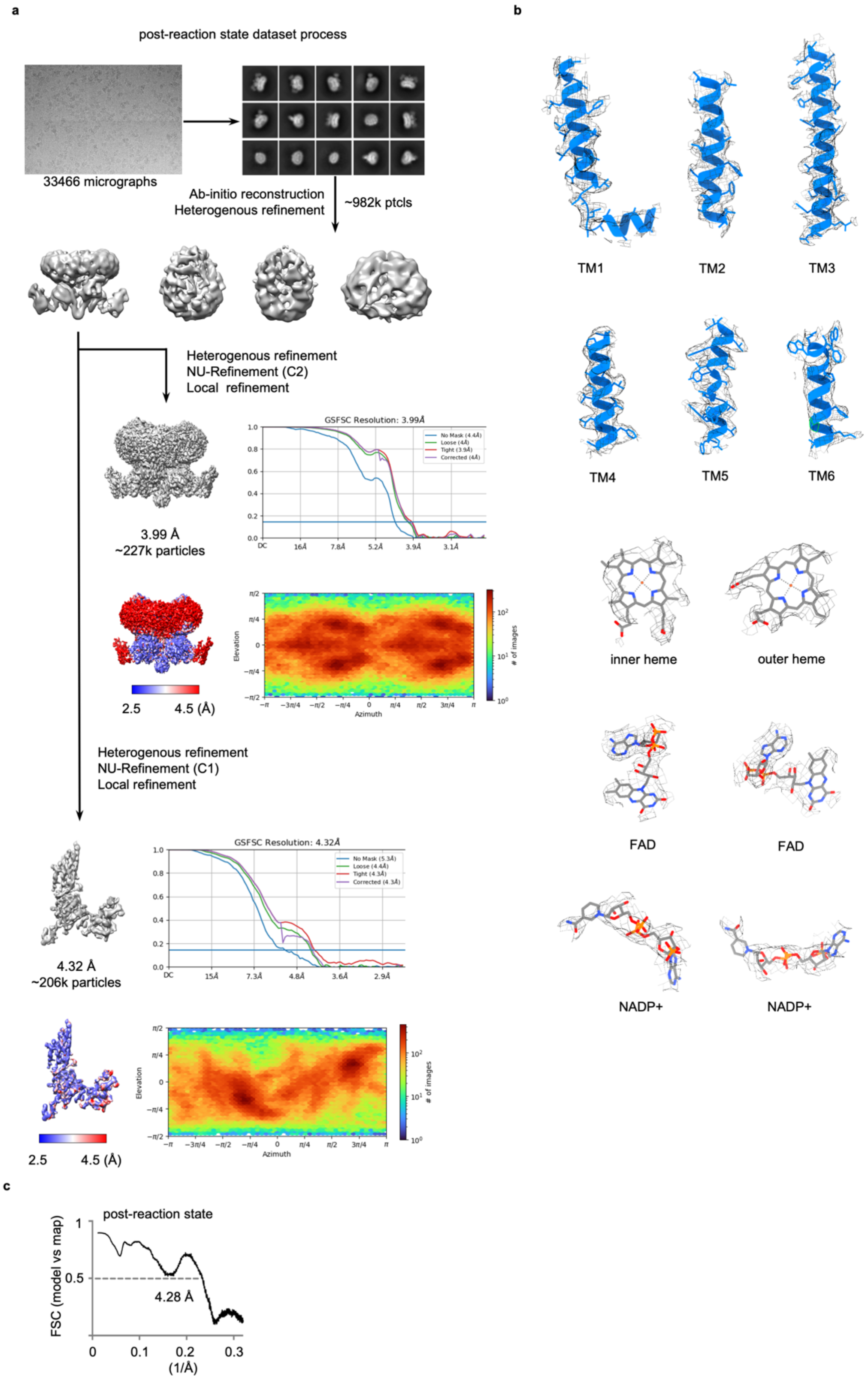
a
post-reaction state dataset process
33466 micrographs
Ab-initio reconstruction
Heterogenous refinement
~982k ptcls
Heterogenous refinement
NU-Refinement (C2)
Local refinement
3.99 Å
~227k particles
GSFSC Resolution: 3.99Å
2.5
4.5 (Å)
Heterogenous refinement
NU-Refinement (C1)
Local refinement
4.32 Å
~206k particles
GSFSC Resolution: 4.32Å
2.5
4.5 (Å)
b
TM1
TM2
TM3
TM4
TM5
TM6
inner heme
outer heme
FAD
FAD
NADP+
NADP+
c
post-reaction state
FSC (model vs map)
4.28 Å
(1/Å)

Supplementary Figure 3 Cryo-EM image analysis of NOX5 in post-reaction state and representative cryo-EM density maps. **a.** Cryo-EM data processing flow of NOX5 in post-reaction state (with NADP+ and Ca^{2+}). **b.** The cryo-EM densities and models of transmembrane helices and small molecules. **c.** Fourier shell correlation (FSC) curves between model and map of NOX5 in post-reaction state.

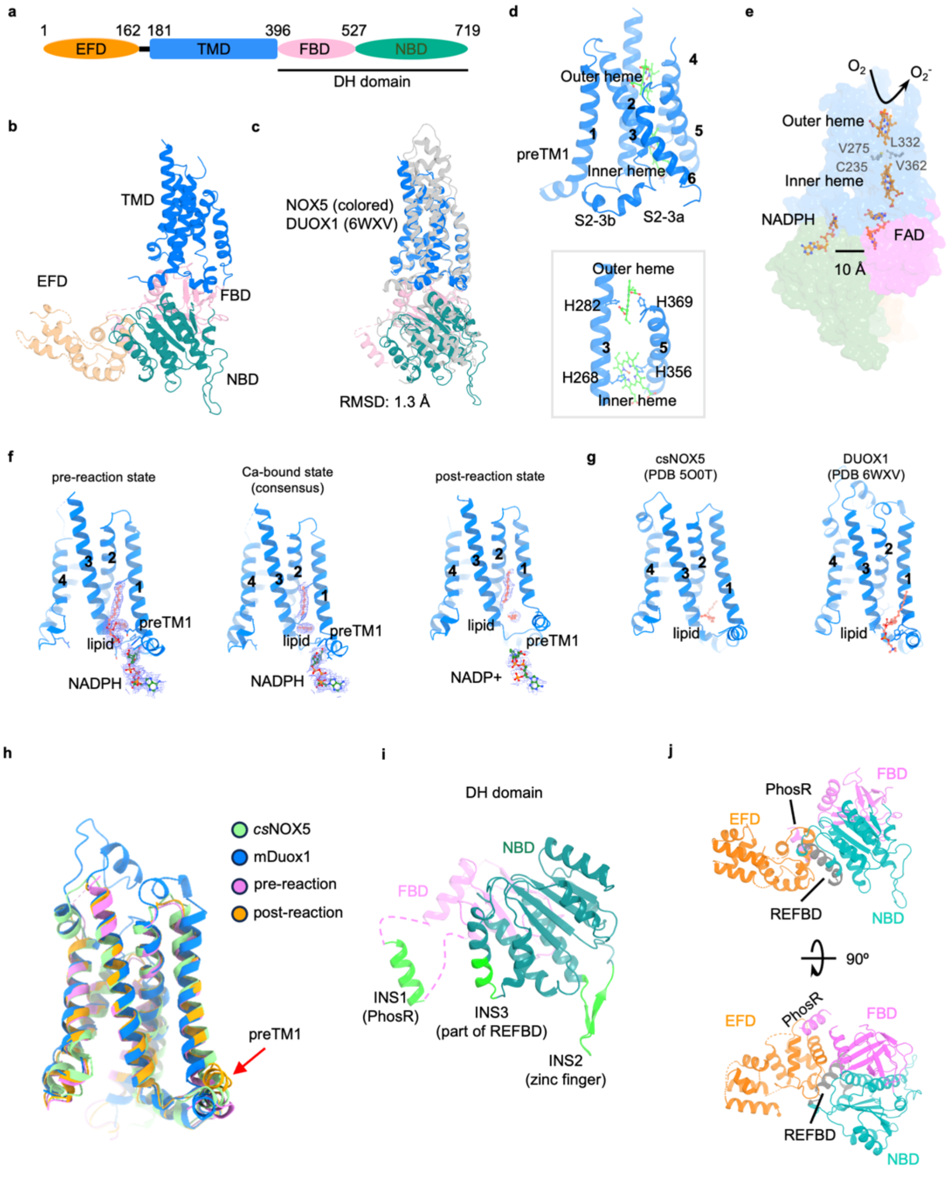

Supplementary Figure 4 Structural features of NOX5. **a.** Domain architecture of human NOX5. **b.** Protomer structure of human NOX5. **c.** Structural comparison between human NOX5 and DUOX1 catalytic cores. NOX5 is colored and DUOX1 is in grey. **d.** The transmembrane domain of human NOX5 and the heme-coordinating residues. **e.** The electron transfer pathway in NOX5: NADPH – FAD – inner heme – outer heme - oxygen. Side chains of residues between two hemes are shown. **f.** The cryo-EM densities of lipid and NADPH for pre-reaction state, intermediate state

(consensus model) and post-reaction state. Lipid or alkyl chains are colored in red. NADPH or NADP+ is colored in green. **g.** The lipid-binding pocket of *cs*NOX5 and mouse DUOX1. Lipid or alkyl chains are colored in red. **h.** Structural comparison of preTM1 orientation between *cs*NOX5, mouse DUOX1 and human NOX5. *cs*NOX5 is colored in pale green. Mouse DUOX1 is colored in blue. Pre-reaction and post-reaction states of human NOX5 are colored in magenta and orange, respectively. PreTM1 is indicated with red arrow. **i.** Unique sequence insertions in the DH domain. **j.** Structural details of the cytosolic DH domain.

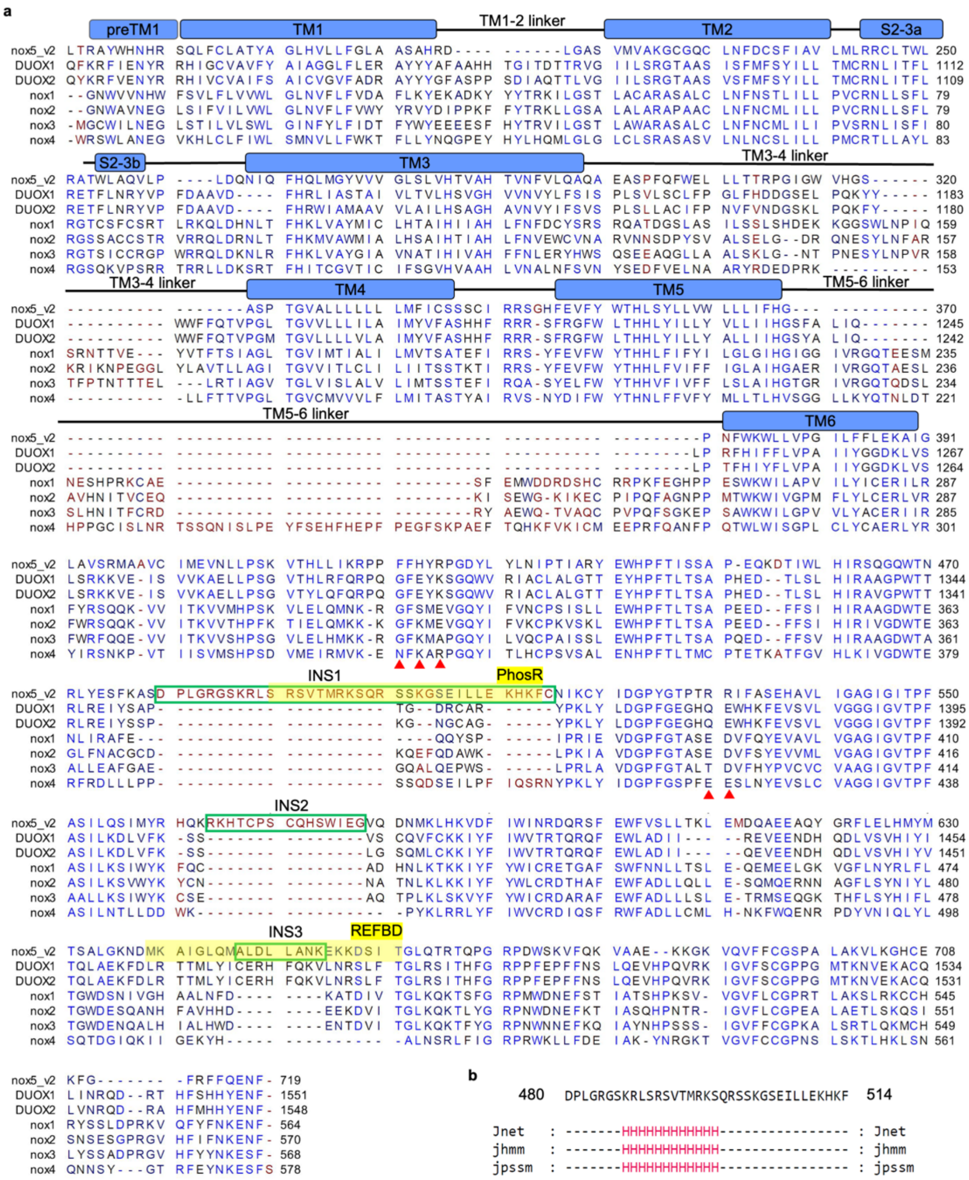

Supplementary Figure 5 Sequence analysis of NOX5. **a.** Sequence alignment of NOX5 paralogs. Transmembrane helices are labeled as TM1-6. Sequence in green boxes are unique insertions (INS1-3) in the DH domain. PhosR and REFBD are highlighted. Dimer interface residues are indicated by red triangles and last residue of NOX4 is indicated by a red color. **b.** Secondary structure predication of the PhosR segment by the Jpred server[1].

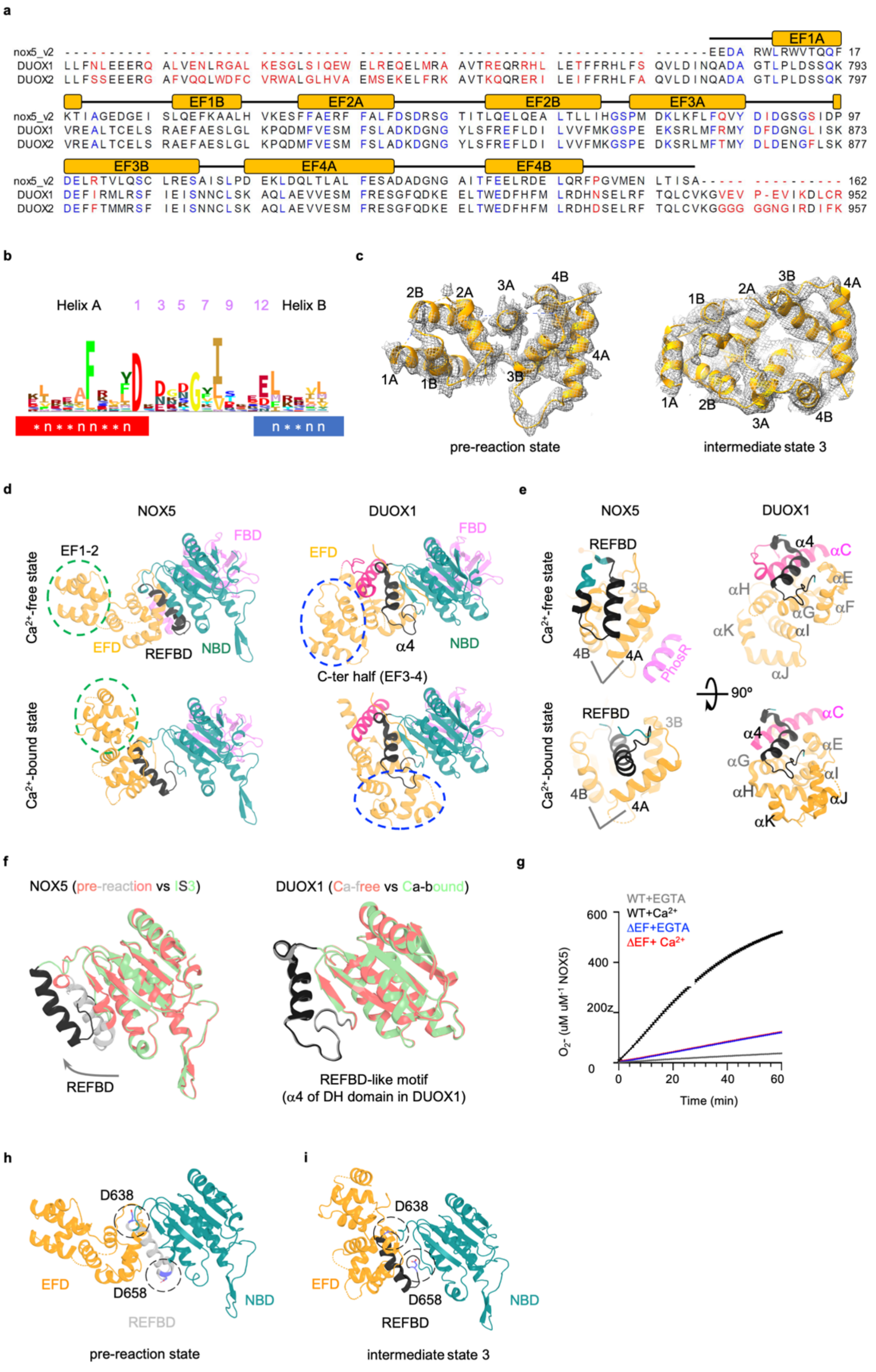
a
EF1A
nox5_v2 ---------- ---------- ---------- ---------- ---------- ---------- ------EEDA RWLRWVTQQF 17
DUOX1 LLFNLEEERQ ALVENLRGAL KESGLSIQEW ELREQELMRA AVTREQRRHL LETFFRHLFS QVLDINQADA GTLPLDSSQK 793
DUOX2 LLFSSEEERG AFVQQLWDFC VRWALGLHVA EMSEKELFRK AVTKQQRERI LEIFFRHLFA QVLDINQADA GTLPLDSSQK 797
EF1B EF2A EF2B EF3A
nox5_v2 KTIAGEDGEI SLQEFKAALH VKESFFAERF FALFDSDRSG TITLQELQEA LTLLIHGSPM DKLKFLFQVY DIDGSGSIDP 97
DUOX1 VREALTCELS RAEFAESLGL KPQDMFVESM FSLADKDGNG YLSFREFLDI LVVFMKGSPE EKSRLMFRMY DFDGNGLISK 873
DUOX2 VREALTCELS RAEFAESLGL KPQDMFVESM FSLADKDGNG YLSFREFLDI LVVFMKGSPE DKSRLMFTMY DLDENGFLSK 877
EF3B EF4A EF4B
nox5_v2 DELRTVLQSC LRESAISLPD EKLDQLTLAL FESADADGNG AITFEELRDE LQRFPGVMEN LTISA----- ---------- 162
DUOX1 DEFIRMLRSF IEISNNCLSK AQLAEVVESM FRESGFQDKE ELTWEDFHFM LRDHNSELRF TQLCVKGVEV P-EVIKDLCR 952
DUOX2 DEFFTMMRSF IEISNNCLSK AQLAEVVESM FRESGFQDKE ELTWEDFHFM LRDHDSELRF TQLCVKGGGG GGNGIRDIFK 957
b
Helix A 1 3 5 7 9 12 Helix B
*n**nn**n
n**nn
c
2B 2A 3A 4B 1A 1B 3B 4A
pre-reaction state
1B 2A 3B 4A 1A 2B 3A 4B
intermediate state 3
d
NOX5
DUOX1
Ca²⁺-free state
Ca²⁺-bound state
EF1-2
FBD
EFD
REFBD
NBD
α4
C-ter half (EF3-4)
e
NOX5
DUOX1
REFBD
3B
4B
4A
PhosR
α4
αC
αE
αF
αH
αG
αI
αK
αJ
90°
f
NOX5 (pre-reaction vs IS3)
DUOX1 (Ca-free vs Ca-bound)
REFBD
REFBD-like motif
(α4 of DH domain in DUOX1)
g
WT+EGTA
WT+Ca²⁺
ΔEF+EGTA
ΔEF+ Ca²⁺
O₂⁻ (uM uM⁻¹ NOX5)
600
400
200
0
0 20 40 60
Time (min)
h
D638
D658
EFD
REFBD
NBD
pre-reaction state
i
D638
D658
EFD
REFBD
NBD
intermediate state 3

Supplementary Figure 6 Conformational changes in EFD. **a.** Sequence alignment of the EFD. The EF hands are labeled as EF1A-EF1B to EF4A-EF4B. **b.** Canonical sequence of an EF hand. Helix A and Helix B are shown. Residue numbers of loop are indicated with 1, 3, 5, 7, 9 and 12. **c.** Cryo-EM densities of EFD with pre-reaction state and intermediate state 3. **d-e.** Comparison of the EFD between NOX5 and DUOX1 upon Ca^{2+} binding. **f.** The movement of REFBD upon activation in NOX5 and DUOX1. **g.** Impact of EFD on the enzymatic activity of NOX5. Curved are plotted (average +/-SD) with 5 technical repeats. Y-axis indicates the generation rate of O_2^- (μM/μM (NOX5)), x-axis indicates the time course (min). Source data are provided as a Source Data file. **h.** Conserved aspartates (D638 and D658) locate in REFBD with pre-reaction state. **i.** Conserved aspartates (D638 and D658) locate in REFBD with intermediate state 3.

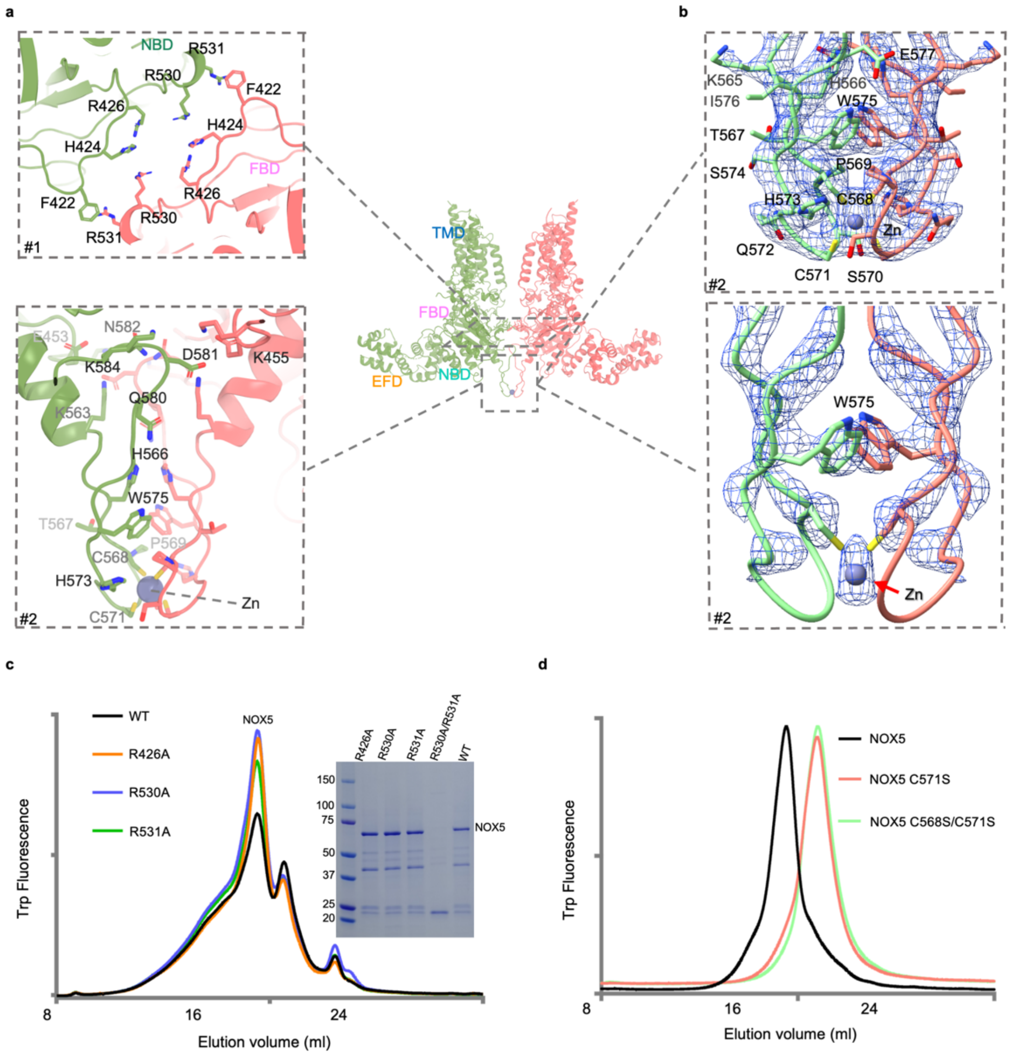

Supplementary Figure 7 Dimer interface of NOX5. **a.** Structural details of NOX5 dimer interfaces #1 and #2. **b**. The cryo-EM density of the dimer interface #2 near the zinc-binding site. Top panel and bottom panel show the cryo-EM densities of interface #2 near the zinc-binding site with level threshold values of 0.58 and 0.88 (in ChimeraX), respectively. Zinc density is indicated with red arrow. **c.** Gel filtration curves of NOX5 and interface mutants (R426A, R530A and R531A) using a Superose 6 increase column by monitoring Trp fluorescence. SDS-PAGE of the purified constructs are shown. We couldn't purify the R530A/R531A double mutation. Source data are provided as a Source Data file. **d.** Gel filtration curves of NOX5 and putative zinc-binding cysteine

mutants (C571S and C568S/C571S) using a Superose 6 increase column by monitoring Trp fluorescence. We couldn't purify the C568S mutation. Source data are provided as a Source Data file.

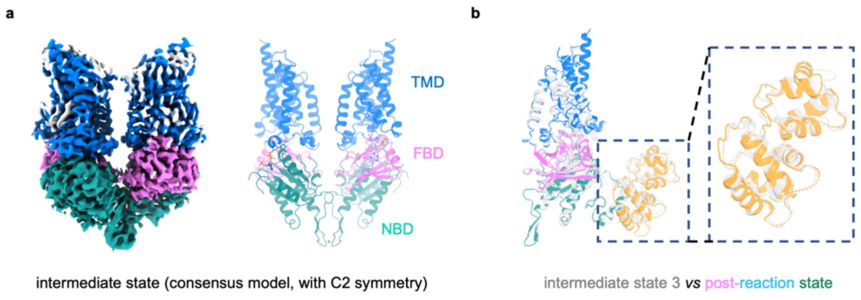

Supplementary Figure 8 Consensus structure of the intermediate state. **a.** Cryo-EM map (left panel) and atomic model (right panel) of the NOX5 intermediate state (consensus model, with C2 symmetry) are shown. **b.** Structural comparison of NOX5 protomers between post-reaction (colored) state and intermediate state 3 (grey).

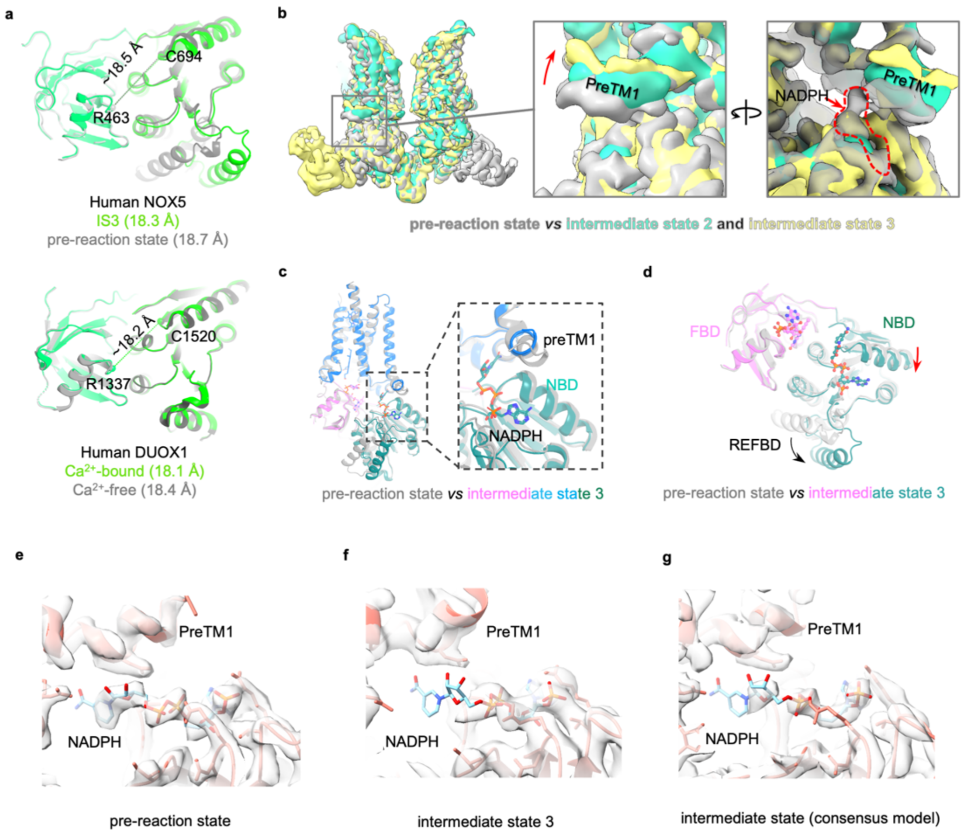

Supplementary Figure 9 Structural comparison in the catalytic core of NOX5 before and after Ca^{2+} activation. **a.** Distance between NBD and FBD in human NOX5 and DUOX1 structures. Pre-reaction state and intermediate 3 of NOX5 are colored in grey and green, respectively. Ca^{2+}-free and Ca^{2+}-bound structures of DUOX1 are colored in grey and green, respectively. **b.** The movement of preTM1 densities between pre-reaction state and intermediate states. Pre-reaction state, intermediate state 2 and intermediate state 3 are colored in grey, cyan and yellow, respectively. **c.** Structural alignment of the NOX5 catalytic cores with pre-reaction state and intermediate state 3. The NADPH-binding site is zoomed in to show the movement of preTM1 relative to the rest. **d.** The subtle movement of NBD in response to the REFBD displacement. **e.** The local cryo-EM density near NADPH of pre-reaction state. **f.** The local cryo-EM density near

NADPH of intermediate state 3. **g.** The local cryo-EM density near NADPH of intermediate state (consensus model).

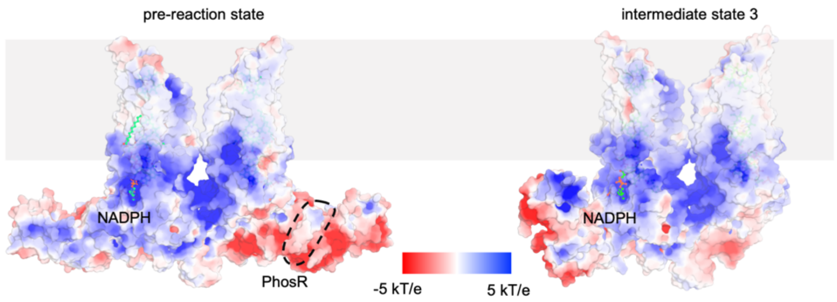

Supplementary Figure 10 Electrostatic surface representation of NOX5 in pre-reaction state and intermediate state 3. The PhosR segment is indicated by a black dashed cycle.

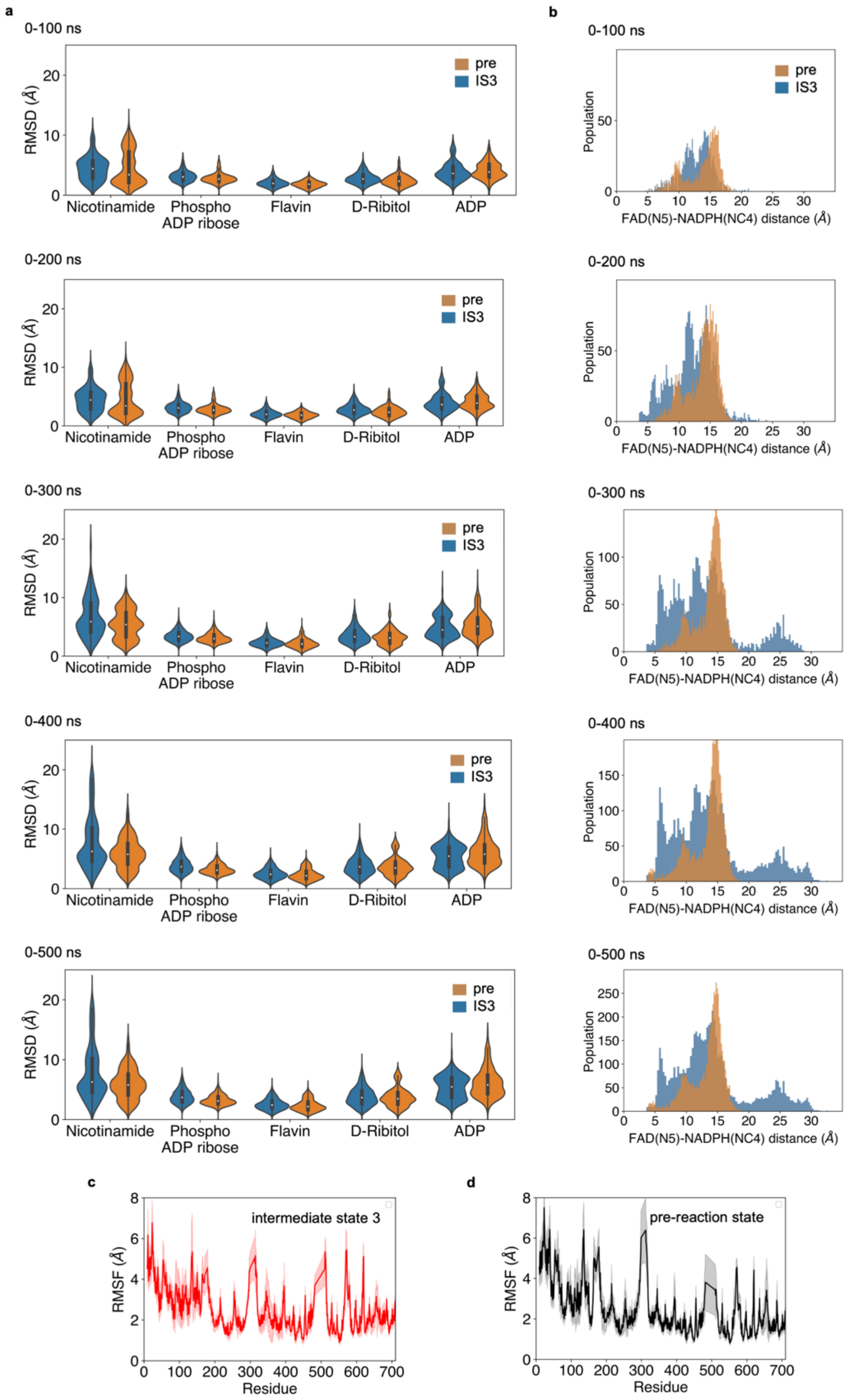
a
0-100 ns
0-200 ns
0-300 ns
0-400 ns
0-500 ns
RMSD (Å)
pre
IS3
Nicotinamide
Phospho ADP ribose
Flavin
D-Ribitol
ADP
b
Population
FAD(N5)-NADPH(NC4) distance (Å)
c
intermediate state 3
RMSF (Å)
Residue
d
pre-reaction state

Supplementary Figure 11 Fractions of MD simulation. **a.** The RMSD of chemical groups from NADPH and FAD in pre-reaction state and intermediate state 3 at every 100 ns up to 500 ns, showing data convergence. The minima, maxima, centre, 25% percentile and 75% percentile of groups are provided with Supplementary Table 3. The figure of 0-500 ns is the same as Figure 3e and the minima, maxima, centre, 25% percentile and 75% percentile values are described in legend. The bar covers 25% and 75% of the data. All data are plotted on the violin plot. Pre and IS3 represent pre-reaction state and intermediate state 3, respectively. Data in all triplicates are combined to a single histogram. **b.** Distance distribution between FAD and NADPH in pre-reaction state and intermediate state 3 at every 100 ns up to 500 ns, showing data convergence. Data in all triplicates are combined to a single histogram. Pre and IS3 represent pre-reaction state and intermediate state 3, respectively. **c.** The root mean square fluctuation (RMSF) analysis of NOX5 amino acid side chains with intermediate state 3 (red) over 500 ns (n=6). **d.** The root mean square fluctuation (RMSF) analysis of NOX5 amino acid side chains with pre-reaction state (black) over 500 ns (n=6). The shaded region shows the standard deviation. Data in all triplicates are combined to a single histogram.

Supplementary Tables

Supplementary Table 1 Cryo-EM data collection, refinement and validation statistics.

	NOX5 (EGTA-NADPH) (EMDB-42014) (PDB 8U85)	NOX5 (Ca-NADPH)				NOX5 (Ca-NADP+) (EMDB-42013) (PDB 8U7Y)
		Consensus (EMDB-42015) (PDB 8U86)	Ca state 1	Ca state 2	Ca state 3 (EMDB-42016) (PDB 8U87)	
**Data collection and processing**						
Magnification	105,000	105,000				130,000
Voltage (kV)	300	300				300
Electron exposure (e–/Å^2)	82.6	81.8				76.886
Defocus range (μm)	0.8~1.6	0.8~1.6				1~2.6
Pixel size (Å)	0.826	0.826				0.6485
Symmetry imposed	C2	C2	C1	C1	C1	C2
Initial particle images (no.)	1,373,175	2,001,493				2,058,434
Final particle images (no.)	83,823	201,424	60,435	66,111	89,751	220,000
Map resolution (Å)	3.2	3.3	4.31	4.03	3.86	4.06
FSC threshold	0.143	0.143	0.143	0.143	0.143	0.143
Map resolution range (Å)	2.5-30	2.5-30	3.5-30	3-30	3-30	3-30
**Refinement**						
Initial model used (PDB code)	5O0T 5O0X					
Model resolution (Å)	3.3	3.5	N/A	N/A	3.8	4.3
FSC threshold	0.5	0.5			0.5	0.5
Model resolution range (Å)	3.3-30	3.5-30			3.8-30	4.3-30
Map sharpening *B* factor (Å^2)	-118	-140	-136	-160	-135	-172
Model composition						
Non-hydrogen atoms	10203	7623			8575	8803
Protein residues	719	719			719	719
Ligands	HEB (2), NDP, FAD, PX2, ZN	HEB (2), NDP, FAD, D12, D10, ZN			HEB (2), NDP, FAD, D12, ZN	HEC (2), NAP, FAD, D12, D10, ZN
B factors (Å^2)						
Protein	65.32	27.45			80.59	80.96
Ligand	37.37	20.23			68.86	52.80
R.m.s. deviations						
Bond lengths (Å)	0.009	0.006	N/A	N/A	0.005	0.003
Bond angles (°)	0.788	0.704			0.643	0.639
Validation						
MolProbity score	1.94	2.03	N/A	N/A	1.98	1.80
Clashscore	11.24	11.72			12.80	7.36
Poor rotamers (%)	0.61	0.79			0.00	0.00
Ramachandran plot						
Favored (%)	94.51	92.92	N/A	N/A	94.73	94.19
Allowed (%)	5.49	7.08			5.27	5.81
Disallowed (%)	0.00	0.00			0.00	0.00

Supplementary Table 1 Cryo-EM data collection, refinement and validation statistics.

Supplementary Table 2 System set-up detail.

System	Box dimension	NOX5	Heme-B	NADPH	FAD	Zn	POPC	Na^+	Cl^-	TIP3P water
Pre-reaction state	15.8 x 15.8 x 13.9 nm^3	2	4	2	2	1	735	220	216	78739
Intermediate state 3	15.8 x 15.8 x 13.7 nm^3	2	4	2	2	1	733	218	214	77602

Supplementary Table 2 System set-up detail.

Supplementary Table 3 Maxima, Minima and percentile values of Supplementary Figure 11a.

	Intermediate state 3					Pre-reaction state				
Group	Centre	Maxima	Minima	75% percentile	25% percentile	Centre	Maxima	Minima	75% percentile	25% percentile
0-100 ns										
Nicotinamide	4.42	11.91	0.00	5.76	2.90	3.42	12.96	0.03	7.24	2.15
Phospho-ADP-ribose	3.07	6.64	0.00	3.67	2.46	2.70	6.22	0.03	3.17	2.29
Flavin	1.97	4.64	0.00	2.43	1.57	1.83	3.66	0.04	2.20	1.41
D-ribitol	2.71	6.65	0.00	3.38	2.18	2.34	6.00	0.03	2.94	1.79
ADP	3.66	9.33	0.00	4.79	2.89	3.88	8.53	0.04	5.11	3.10
0-200 ns										
Nicotinamide	5.13	19.67	0.00	6.65	3.50	5.10	12.96	0.03	7.45	2.84
Phospho-ADP-ribose	3.15	6.64	0.00	3.71	2.61	2.89	7.44	0.03	3.41	2.44
Flavin	2.11	4.64	0.00	2.52	1.71	1.91	4.00	0.04	2.33	1.50
D-ribitol	2.98	7.43	0.00	3.77	2.41	2.76	6.00	0.03	3.38	1.98
ADP	4.16	9.84	0.00	5.57	3.23	4.91	11.33	0.04	6.04	3.56
0-300 ns										
Nicotinamide	5.90	21.11	0.00	9.10	4.14	5.38	12.96	0.03	7.43	3.30
Phospho-ADP-ribose	3.35	7.88	0.00	4.01	2.76	3.00	7.44	0.03	3.62	2.50
Flavin	2.26	5.49	0.00	2.77	1.80	2.08	6.13	0.04	2.65	1.62
D-ribitol	3.31	9.17	0.00	4.33	2.50	3.13	8.56	0.03	3.92	2.20
ADP	4.50	13.74	0.00	6.60	3.39	5.10	13.98	0.04	6.54	3.85
0-400 ns										
Nicotinamide	6.29	22.51	0.00	9.95	4.22	5.58	15.07	0.03	7.85	3.64
Phospho-ADP-ribose	3.55	8.13	0.00	4.40	2.87	3.07	7.44	0.03	3.76	2.60
Flavin	2.32	5.68	0.00	2.95	1.78	2.16	6.13	0.04	2.86	1.64
D-ribitol	3.47	9.17	0.00	4.59	2.58	3.37	8.96	0.03	4.28	2.44
ADP	5.07	13.74	0.00	6.91	3.48	5.55	13.98	0.04	7.10	4.20

Supplementary Table 3 Maxima, Minima and percentile values of Supplementary Figure 11a.

Supplementary References

1 Drozdetskiy, A., Cole, C., Procter, J. & Barton, G. J. JPred4: a protein secondary structure prediction server. Nucleic acids research 43, W389-W394 (2015).
