# Supplementary material for: Structural basis of human NOX5 activation
Source: Nat Commun. 2024 May 11;15:3994. doi: 10.1038/s41467-024-48467-y (PMC11088703; doi:10.1038/s41467-024-48467-y)
Supplement: Supplementary file 3 — Description of Additional Supplementary Files [file 41467_2024_48467_MOESM3_ESM.pdf]

Description of Additional Supplementary Files

File Name: Supplementary Movie 1
Description: Ca^{2+}-dependent conformational changes of NOX5 from pre-reaction to intermediate state 3. TMD, FBD, NBD and EFD are colored in blue, pink, dark green, and orange, respectively.
